# Environmental influences on mosquito foraging and integrated vector management can delay the evolution of behavioral resistance

**DOI:** 10.1111/eva.12354

**Published:** 2016-01-29

**Authors:** Chris Stone, Nakul Chitnis, Kevin Gross

**Affiliations:** ^1^ Department of StatisticsNorth Carolina State UniversityRaleighNCUSA; ^2^ Swiss Tropical and Public Health InstituteBaselSwitzerland; ^3^ University of BaselBaselSwitzerland

**Keywords:** *Anopheles*, attractive toxic sugar baits, behavioral resistance, foraging behavior, integrated vector management, long‐lasting insecticidal bed nets, malaria, model, mosquito

## Abstract

Along with the scaled‐up distribution of long‐lasting insecticidal nets for malaria control has become concern about insecticide resistance. A related concern regards the evolution of host‐seeking periodicity from the nocturnal to the crepuscular periods of the day. Why we observe such shifts in some areas but not others and which methods could prove useful in managing such behavioral resistance remain open questions. We developed a foraging model to explore whether environmental conditions affect the evolution of behavioral resistance. We looked at the role of the abundance of blood hosts and nectar sources and investigated the potential of attractive toxic sugar baits for integrated control. Higher encounter rates with hosts and nectar sources allowed behaviorally resistant populations to persist at higher levels of bed net coverage. Whereas higher encounter rates with nectar increased the threshold where resistance emerged, higher encounter rates of hosts lowered this threshold. Adding sugar baits lowered the coverage level of bed nets required to eliminate the vector population. In certain environments, using lower bed net coverage levels together with toxic sugar baits may delay or prevent the evolution of behavioral resistance. Designing sustainable control strategies will depend on an understanding of vector behavior expressed in local environmental conditions.

## Introduction

Following the emergence of drug resistance to chloroquine in many areas, current malaria control largely depends on the use of artemisinin combination therapy and the mass deployment of long‐lasting insecticidal bed nets (LLINs). Particularly the latter has had a dramatic impact on reducing the transmission intensity of malaria (Trape et al. 2011), and as a result, great strides have been made in reducing malaria incidence (O'Meara et al. 2010; WHO, 2014).

Long‐lasting insecticidal bed nets reduce covered humans' exposure to bites by the nocturnal anopheline vectors of *Plasmodium* spp. These pyrethroid‐impregnated nets can also kill mosquitoes upon contact. Given the tight linkage between a mosquito's ability to obtain blood meals and her reproductive fitness, a seemingly inevitable consequence of the mass rollout of LLINs is the emergence of insecticide resistance (Koella et al. 2009; Read et al. 2009). Pyrethroid resistance has indeed increased and is now widespread across Africa (Ranson et al. 2011). Insecticide resistance to pyrethroids takes multiple forms, such as metabolic resistance (the overproduction of enzymes that sequester or detoxify the insecticide), or target site resistance (which reduces the neurotoxic efficacy of the insecticide) (Kelly‐Hope et al. 2008). While the implications for malaria transmission of the spread of resistance are not well understood (Rivero et al. 2010; Ranson et al. 2011), it has been estimated that it could lead to an additional 120 000 deaths per year (WHO, 2012), and may interfere with the prospects for sustained control or the feasibility of achieving malaria elimination.

Exacerbating these concerns is that pyrethroids are currently the only class of insecticide approved for impregnation of bed nets. Due to the absence of alternative approved insecticides, management and prevention of the further spread of resistant genotypes is currently our most promising option. However, this may be hampered by the lack of access to the traditional methods of resistance management used in agriculture, which rely heavily on rotation or mosaics of different insecticides. Hence, there is strong interest in novel control interventions designed to be evolution‐proof or at least able to significantly delay the emergence of insecticide resistance (Koella et al. 2009; Read et al. 2009).

A further complication for the development of resistance management strategies is that in addition to physiological resistance, mosquito behavior could evolve in a manner to diminish the impact of LLINs. Such behavioral resistance could consist of a change in host‐seeking behavior that results in a greater proportion of bites being taken on unprotected hosts. This could occur if mosquitoes bit closer to dusk or dawn instead of in the middle of the night, or outdoors rather than indoors. Conceivably, preferences for host types could also shift, so that non‐human animals are targeted for a blood meal more frequently (Lefèvre et al. 2009). And additionally, some authors have speculated that life histories that favor an increase in early life fecundity over longevity could also be selected for by vector control programs (Ferguson et al. 2012). While the latter two could be beneficial and lead to reduced transmission, this may not be the case for a shift from nocturnal to crepuscular feeding. Modeling studies suggest that the impact of behavioral resistance on the efficacy of control interventions may be of the same magnitude as that of physiological resistance (Briët and Chitnis 2013; Gatton et al. 2013).

To date, the evidence for behavioral resistance developing in vector populations following the deployment of LLINs remains scarce (Gatton et al. 2013). While distinct shifts in peak biting times have been observed (Russell et al. 2011; Moiroux et al. 2012), other studies have reported that exposure to bites during the times most humans were asleep remained the norm (Huho et al. 2013), even when a shift in peak biting times did occur (Bayoh et al. 2014). Interpreting these results can be difficult, because shifts to early evening or morning biting could result not just from the evolution of diel periodicity, but can also represent behavioral or phenotypic plasticity (e.g., extended foraging bouts or learning). In some instances, primary vectors such as *Anopheles gambiae sensu stricto* Giles appear to be replaced by anopheline species with more plastic behaviors, such as *Anopheles arabiensis* Patton. Given these confounding factors, it is perhaps not surprising that the evidence for shifts in periodicity has been equivocal. Open questions are why we find particular ecological or evolutionary responses in mosquito populations to control interventions in some localities, but different responses in others; whether plasticity in foraging behavior promotes or diminishes an adaptive response; and how to manage the evolution of behavioral resistance.

Recent theoretical advances in resistance management have illustrated how consideration of evolutionary theory (e.g., the evolution of senescence) can be employed to design or deploy insecticides in a manner that considerably delays the spread of resistance (Koella et al. 2009; Read et al. 2009). It is also becoming clear that the dynamics of sensitive and resistant mosquitoes depend on both the fitness costs of resistance in the absence of an insecticide, and the fitness benefits of resistance when exposed to the insecticide, and that both these costs and benefits can be modulated by environmental stressors (Koella et al. 2009, 2012). For instance, in *Anopheles* spp. infection with pathogenic fungi as well as a microsporidian restored the sensitivity to insecticides of resistant strains (Farenhorst et al. 2009; Koella et al. 2012). Competitive interactions with other species may also play a role, for instance, in the mosquito *Culex quinquefasciatus*, interspecific competition in the larval stage with *Daphnia magnia* led to a slower rate of spread (Becker and Liess 2015). In the diamondback moth, *Plutella xylostella*, poor nutritional quality or resource limitation acted as an environmental stressor with an effect on the cost of resistance (Raymond et al. 2005). Although poorly understood at the proximate level, it is plausible that these effects are mediated by a resource‐based trade‐off. It has been shown that resistant mosquitoes contain less energetic reserves upon emergence than sensitive ones (Hardstone et al. 2010; Rivero et al. 2011).

Here, we develop and use a mathematical model to investigate whether environmental factors may likewise influence the spread of behavioral resistance. Because the costs and benefits of behavioral resistance are likely determined by different factors or trade‐offs than those governing physiological resistance, it is not obvious whether environmental stressors will have similar delaying effects. We focus on environmental aspects directly related to mosquito foraging: the abundance of nutrition in the form of blood hosts and nectar sources, and hypothesize that the spread of a behaviorally resistant genotype with a fixed crepuscular feeding pattern may be accelerated in harsher environments with fewer resources. This could result from, for instance, behavioral plasticity in sensitive mosquitoes, (i.e., the ability to continue foraging after encountering a protected host and obtaining a blood meal elsewhere later that night or early the following evening) being greater in environments where sugar can be taken to facilitate longer foraging flights (Stone et al. 2012a). In this case, greater plasticity in foraging behavior will diminish the fitness advantages associated with an evolved crepuscular foraging pattern. Alternatively, in areas with dense populations of hosts, a nocturnal foraging pattern may still be advantageous if searching for different (uncovered) hosts requires only short foraging bouts. Thus, the type of behavioral plasticity we consider here relates to plasticity in the duration of foraging bouts, when the underlying host‐seeking schedules or periodicities are not altered.

In addition, we also explore the ramifications of deploying multiple vector control methods on the evolution of behavioral resistance. The combined deployment of LLINs and a complementary method provides an example of integrated vector management (IVM), an approach that guides the selection of vector control interventions, based on knowledge of local ecological conditions (van den Berg et al. 2013). IVM aims to improve cost‐effectiveness and long‐term sustainability, and has been proposed as a tool in insecticide resistance management (Thomas et al. 2012). It would manage resistance by simultaneously deploying two or more vector control methods that complement each other and target different behaviors or life stages of the vector. Developing or utilizing such novel vector control tools is also a priority for areas where residual transmission occurs, for instance following the development of behavioral resistance (Killeen 2014). Evidence of the efficacy of IVM as a resistance management strategy, particularly for behavioral resistance, however, is lacking. Additionally, the extent to which the efficacy of IVM methods depends on environmental factors is an understudied area. One example which has been suggested to have potential for an IVM approach in combination with LLINs are attractive toxic sugar baits (ATSB) (Müller et al. 2010; Marshall et al. 2013; Stewart et al. 2013). ATSB are composed of a sugary solution, which is either deployed within a bait station or sprayed on vegetation. The solution further consists of an olfactory attractant and an oral insecticide, such as boric acid. We use ATSB as a practical example to explore the possibilities of IVM as a resistance management strategy and the extent to which environmental factors influence its efficacy.

## Model

### Structure and assumptions

We develop a periodic projection matrix model (Caswell 2001) of the mosquito feeding cycle, add vector control interventions, and analyze this model for two parameterizations representing nocturnal and crepuscular (i.e., behaviorally resistant) populations of mosquitoes. We employ this approach as it achieves a good balance between added behavioral and physiological complexity of adult vectors, which may be particularly relevant for understanding the evolution and impact of behavioral resistance, while still allowing for a tractable model. We use the model to investigate under which conditions (both varying certain environmental factors and the coverage levels of control interventions) behaviorally resistant mosquitoes are favored. We assume that, all else being equal and given sufficient time, resistance will emerge if the fitness (the intrinsic rate of increase, *λ*) of resistant mosquitoes is greater than that of sensitive mosquitoes.

Our model makes the following simplifying assumptions: (i) We ignored any possible density‐dependent effects operating on the immature stages; thus, we assume that factors such as the drying up of the ephemeral breeding sites favored by anophelines, or predator or pathogen‐induced mortality, overshadow any possible density dependence; (ii) We ignore the effects of seasonality; (iii) We assume behavioral resistance is under genetic control and can be selected for, but do not include genetic structure in our model; (iv) We assume the vector in question is entirely anthropophilic, and do not explicitly consider non‐human hosts in this model; (v) We do not allow for the possibility of other types of resistance or behavioral shifts to evolve. These factors are all likely important in reality, and deserving of further study in their own right. For instance, while density dependence likely operates, the magnitude of its effects and the manner through which it operates under natural conditions remain unclear. And whether host‐seeking periodicity is under the control of one, a few, or very many genes is not known (Gatton et al. 2013). Thus, for simplicity and clarity we ignore these factors for now and focus on asymptotic outcomes and proportional changes.

**Figure 1:**
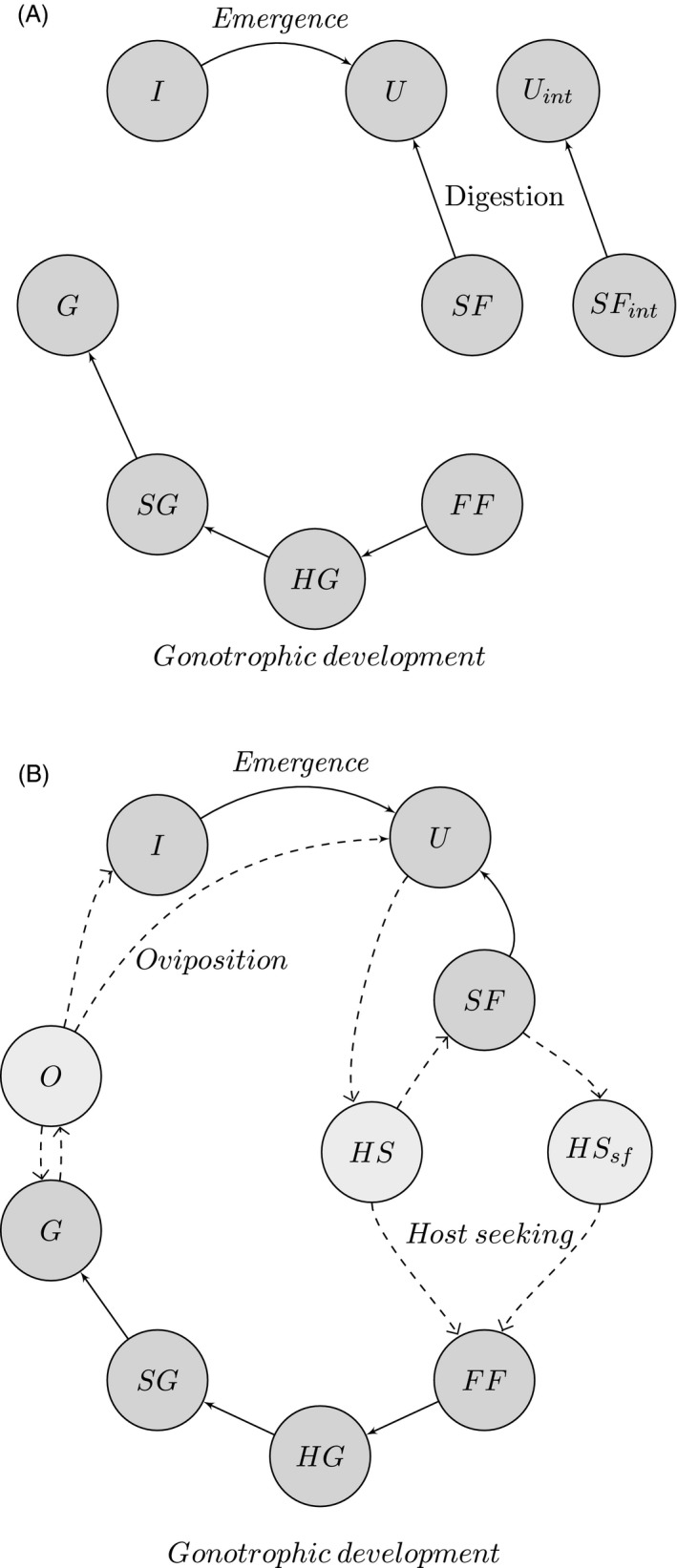
(A) A diagram of the mosquito population characterized by physiological state during daylight hours, where transitions between states correspond to physiological and larval development, but transitions due to foraging or oviposition do not occur (representing the transition matrix, ***M***_*d*_). All states allow self‐transitions which are not shown. (B) The mosquito population characterized by physiological and behavioral states during nighttime hours. Dashed lines represent behavioral transitions, and solid lines represent physiological transitions (representing transition matrix, ***M***_*i*_ for 1 ≤ *i *≤* *12). All states allow self‐transitions which are not shown.

The periodic projection matrix equation (see Fig. 1 for a life cycle diagram) is of the form:

(1) $$N_{\left(t+1\right)}=M_{dn}M_{12}\ldots M_{1}M_{nd}M_{d}N_{\left(t\right)},$$

where the vector *N*_(*t*)_ consists of the states that occur during daytime:

(2) $$N_{\left(t+1\right)}=M_{dn}M_{12}\ldots M_{1}M_{nd}M_{d}N_{\left(t\right)},$$

The distinction between stages of blood digestion (freshly fed, half‐gravid, subgravid, gravid) is made to introduce a delay between blood‐feeding and oviposition. The unfed and sugar‐fed states appear in the normal and the interrupted (int) form. The latter of these describes mosquitoes that failed to blood feed the previous night and were still host seeking at dawn. We assume that these mosquitoes will rejoin the host‐seeking states at dusk, regardless of whether they are of the nocturnal or crepuscular population, while the regular unfed or sugar‐fed mosquitoes rejoin the host‐seeking populations according to the activation pattern of their genotype.

The matrix, ***M***_*d*_, refers to transitions that occur during the daytime, ***M***_*nd*_ refers to the transitions from day to night, ***M***_*i*_ refers to the hourly nighttime transition (for 1 ≤ *i *≤* *12 where *i* = 1 is 19:00, *i* = 2 is 20:00 and so on with *i* = 12 representing 06:00 the next day), and ***M***_*dn*_ refers to the transitions from night to day. The phase‐specific transition matrices in eqn (1) can be multiplied together to form the matrix ***A***_*n*_, which describes transitions from the beginning of one day to the beginning of the next.

(3) $$A_{n}=M_{dn}M_{12}\ldots M_{1}M_{nd}M_{d}$$

At night, the population vector is expanded to include behavioral states (namely host seeking and oviposition‐site seeking) in addition to physiological states:

(4) $$N_{\left(t+1\right)}=M_{dn}M_{12}\ldots M_{1}M_{nd}M_{d}N_{\left(t\right)},$$

The transition matrices and equations for the matrix transition elements are given in the Appendix. A description of the model parameters is provided (Table 1). An important assumption related to the mathematical specification of this model is that a mosquito in a particular stage is exposed to the mortality operating on that stage for the entire time step. A justification is that the time steps used are short, and that changes in mortality rates due to physiological changes are unlikely to be immediate, for instance, following a blood meal it may take a female some time to locate and fly to a safe resting place, while heavily encumbered with a blood meal.

**Table 1:** Description of parameters. Here the subscript *h* where *h* ∈ {*b*,* s*} represents the host type with *b* representing blood hosts and *s* representing sugar sources

Variable	Description	Value	Dim.	Source
*ε*	Duration of immature stage	240	h	Gimnig et al. (2002); Service (1973)
*μ*_*l*_	Immature stage mortality	0.0126	h^−1^	Service (1971, 1973, 1977)
*μ*_*uf*_	Mortality while unfed	0.0093	h^−1^	Stone et al. (2009)
*μ*_*sf*_	Mortality while sugar‐fed	0.0017	h^−1^	Stone et al. (2012b)
*μ*_*r*_	Mortality while resting	0.0026	h^−1^	Killeen et al. (2007)
*μ*_*f*,*i*_	Mortality while foraging at hour *i*	Varies (see Fig. 2)	h^−1^	Assumed; Saul (2003)
$\mu_{f_{s},i}$	Mortality while sugar‐fed and foraging at hour *i*	Varies	h^−1^	Assumed
*ρ*_*i*_	Host‐seeking activation probability at time *i*	Varies (see Fig. 3b)		Assumed
*ρ*_*o*_	Oviposition‐site seeking activation probability	0.5	–	Assumed
*γ*_*h*_	Encounter rate for host type *h*	Varied	h^−1^	–
*γ*_*o*_	Encounter rate for oviposition sites	0.25	h^−1^	Assumed
*σ*_*h*_	Innate preference for host type *h*	0.9 or varied	–	Killeen et al. (2014)
*δ*_*s*_	Duration of sugar positivity	24	h	Gary (2005)
*δ*_*b*_	Gonotrophic stage duration	16	h	Gillies (1953)
*θ*_*i*_	Usage of bed nets at time *i*	Varies (see Fig. 2)	–	Killeen et al. (2006)
$\phi_{b}$	Population coverage of bed nets	Varied	–	–
*r*	Probability of being repelled by a net	0.6	–	Le Menach et al. (2007)
*F*	Fecundity	20	–	Manda et al. (2007)

### Foraging behavior and mosquito life cycle

**Figure 2:**
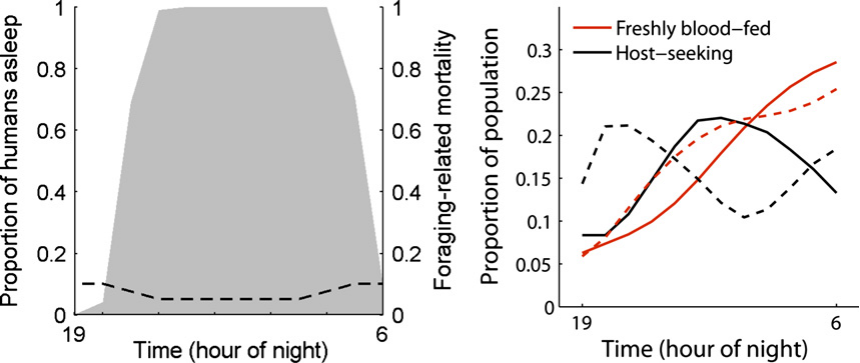
(Left) Foraging‐related mortality (dashed line) and probabilities that humans are asleep by hour of the night (3); (Right) The proportion of freshly fed or host‐seeking adult mosquitoes over the course of the night, for nocturnal (solid lines) and crepuscular (dashed lines) populations.

Unfed mosquitoes will seek blood or nectar meals, and their host‐seeking behavior is guided by innate preference, host availability, and diel periodicity. Here, mosquito foraging is characterized by a general host‐seeking state, which mosquitoes enter during the nighttime based upon their host‐seeking periodicity. Periodic tendencies are incorporated by having activation probabilities associated with each nightly time step, *ρ*_*i*_. These activation probabilities indicate an initiation of host‐seeking behavior, appetitive flights, or increased sensitivity to organic volatile compounds associated with hosts. To our knowledge, these activation patterns have yet to be studied under laboratory or field conditions. Hence, here we assume here that *ρ*_*i*_ varies in different ways over the night according to a nocturnal (increasing from dusk to a peak in the middle of the night, before declining again) or crepuscular genotype (a bimodal pattern) (see Fig. 2). While in the host‐seeking state, a mosquito may encounter a host of a particular type and attempt to feed, or fail to encounter a host and continue with foraging, or succumb to mortality. We assume a host‐seeking mosquito would only choose to cease foraging at dawn. Plasticity in foraging activity is therefore incorporated in the following sense. We are particularly interested in the possibility that shifts in biting activity to dawn or dusk periods could result from mosquitoes (with a nocturnal activation pattern) remaining in a host‐seeking state for longer periods and only biting humans when they emerge from their bed nets. Our model allows for this by assuming that mosquitoes that have entered the host‐seeking state but failed to find a host remain in this state until dawn, and then [following a resting state during the day in an ‘interrupted' state (see Fig. 1)] rejoin the host‐seeking state immediately upon dusk the following day.

Why *Anopheles* spp. have a peak feeding time in the middle of the night, rather than foraging earlier in the evening (which would result in a longer available period to successfully locate a host), is an open question. We assume there is a fitness cost associated with foraging during the crepuscular hours. Such a cost could be due to greater awareness and display of host‐defensive behaviors of vertebrates; predation by vision‐based hunters such as dragonflies; or due to a less hospitable (hotter, drier) environment. To include such a cost, without specifying its cause, we assume mortality associated with foraging, *μ*_*f*,*i*_, is greater in the crepuscular period than the nocturnal period (Fig. 2). We also assume that foraging‐related mortality when sugar‐fed, *μ*_*sf*,*i*_, will be three times as low as *μ*_*f*,*i*_, allowing for a survival advantage related to sugar feeding. A justification is that while sugar‐fed, mosquitoes can sustain longer foraging flights (Kaufmann and Briegel 2004), and foraging mosquitoes may be buffered against the energetic depletion that comes with, and therefore more tolerant to, dehydration (Benoit et al. 2010).

Host choice or exploitation, *a*_*h*_, depends on both the local abundance as well as the innate attractiveness of hosts to mosquitoes. We distinguish between two host types: (i) the major blood host of *Anopheles gambiae s.s*., humans; (ii) all natural nectar sources grouped together as one type. While other animal species, such as cattle, may provide some proportion of blood meals, we do not consider additional host types here (in terms of bed net use, cattle will function as a group of systematic noncompliers, which has implications for disease transmission, but for our fitness‐related purposes, this can reasonably be approximated by varying ITN coverage rates). Further, we assume that all resources are well‐mixed in the environment; that mosquitoes are aware of the relative abundance of resources; and that time or energetic costs associated with entering or leaving houses are negligible. Investigating these issues would require a different type of model (e.g., Roitberg and Mangel 2010), and is beyond the scope of the current paper. Here, host exploitation is given by:

(5) $$a_{h}=\frac{\gamma_{h}\sigma_{h}}{\gamma_{b}\sigma_{b}+\gamma_{s}\sigma_{s}},$$

where *σ*_*h*_ is the relative preference for host type *h*, which is the probability that a mosquito would find an encountered host of that type acceptable, and *γ*_*h*_ is the encounter rate of that host type (where *h* ∈ {*b*, *s*} with *b* representing blood hosts and *s* representing sugar hosts). Following a sugar meal, we assume a mosquito will enter the host‐seeking state again with activation probability *ρ*_*i*_. This delay represents the lowered responsiveness to host stimuli following a sugar meal, which may last for several (ca. 3) hours (Jones and Madhukar 1976).

The manner in which we capture the rest of the mosquito life cycle can briefly be described as follows. We specify a constant mean duration of immature development in hours, *ε*, and assume an exponential rate of development, $\frac{1}{\varepsilon }$. Larval mortality is assumed constant over the immature period, at the hourly rate of *μ*_*l*_. After a mosquito has taken a blood or sugar meal, digestion occurs. Digestion and usage of the reserves acquired through sugar feeding occurs over a period *δ*_*s*_. A blood meal allows for the development of an egg batch. This period, during which a mosquito rests, is marked by the stages of gonotrophic development of average duration *δ*_*b*_. Gravid females seek suitable larval development places to deposit their eggs. They encounter oviposition sites at rate *γ*_*o*_. If ovipositing females encounter a breeding site and survive, they transition back to the unfed stage. At the same rate, a number of eggs, *F*, are deposited into the immature stage.

### Vector control interventions

#### Insecticide‐treated nets

LLINs provide a protective barrier around sleeping humans and repel (through excito‐repellent or irritant properties of the insecticide) or kill a proportion of mosquitoes that contact the net. Without nets, feeding attempts are only unsuccessful due to death related to foraging, *μ*_*f*,*i*_. With nets, a host can be covered by a bed net at a given hour of the night, $\phi_{b,i}.$ This parameter is the product of the probability that those people who possess a net are protected by it at any given hour (*θ*_*i*_) (i.e., they are asleep), and the proportion of the population that possesses a net ($\phi_{b}$),

(6) $$\phi_{b,i}=\phi_{b}\theta_{i}.$$

Thus, mosquitoes can only blood feed on humans who are not protected by LLINs at that hour with the assumption that nets in use are fully effective (they have no holes and mosquitoes cannot bite through them). Previously, remaining in the host‐seeking phase was a function of failing to find a host and surviving the time step. With LLINs, this also has to account for those mosquitoes that encountered a human, made contact with a bed net, and were repelled rather than died.

The behavior of sugar‐fed mosquitoes around bed nets may differ slightly. For instance, in experimental studies sugar‐fed females had a lower responsiveness or attack rate on humans, and spent less time at a host source when it was protected by netting than did sugar‐starved females (Roitberg et al. 2010; Zappia and Roitberg 2012). Although these responses (attack rates, persistence, and diversion to another energetic source) depend nonlinearly on energy reserves (Roitberg et al. 2010), the overall picture is consistent with the notion that energetically richer females will be more risk‐averse, while energetically deprived females will be more risk‐prone (Zappia and Roitberg 2012). We translate these differences in persistence as resulting in different values (indicated by subscript *s*) associated with *r* (a greater probability of leaving the house), and *μ*_*b*_ (a lower probability of dying, due to spending less time in contact with the net), compared to unfed mosquitoes. The insecticidal properties of the net are thus determined by r and *μ*_*b*_ (=1−*r*) as well as the population coverage and usage over the diel, $\phi_{b,i}.$

#### Attractive toxic sugar baits

A mosquito that feeds from a sugar‐baited trap dies. The probability that a sugar meal is taken from a trap rather than a natural source depends on the encounter rates and relative preference for both. Sugar‐baited traps could be construed as bait stations that are added to the environment, or as applications of sugary toxic solutions on plants already in the environment. We distinguish between these versions of ATSB, and refer to the first as sugar bait coverage and the second as nectar source coverage.

Sugar bait coverage depends on the encounter rates $\gamma_{s_{n}}$ and $\gamma_{s_{b}}$, and the preferences $\sigma_{s_{n}}$ and $\sigma_{s_{b}}$, where the subscript, *s*_*n*_ denotes natural sugar sources and *s*_*b*_ denotes artificial sugar baits. The effective coverage of sugar‐baited traps then represents the probability of encountering and feeding on a bait rather than a natural sugar source: $\phi_{sb}=\frac{\gamma_{s_{b}}\sigma_{s_{b}}}{\gamma_{s_{n}}\sigma_{s_{n}}+\gamma_{s_{b}}\sigma_{s_{b}}}$. In contrast, the alternative method where existing nectar sources are replaced by toxic ones, for instance by spraying a sugary solution on them, simply assumes that a proportion of the nectar sources, $\phi_{s}$ will be toxic.

#### Demographic outcomes

As with nonperiodic matrix models, the intrinsic rate of increase, *λ*, can be obtained by finding the dominant eigenvalue of the transition matrix, ***A***_*n*_, and the stable age distribution of the population is given by the right eigenvector of ***A***_*n*_ (Caswell 2001). The latter can be used to determine the proportion of adults that are in the host‐seeking state over the course of the night (Fig. 2).

A metric more closely tied to epidemiological outcomes is required to investigate the consequences of behavioral resistance for the efficacy of vector control programs. We use the number of bites females are expected to make over the course of their lifetime, rather than use a measure such as vectorial capacity (Dye 1992), as the model is not well suited to predict changes in population size. This metric is comparable to the stability index of malaria and can be thought of as one aspect of vectorial capacity, namely the expected number of infective bites that could arise from one vector, conditional on having survived the incubation period of the parasite (Smith and McKenzie 2004).

To find the expected number of lifetime bites per mosquito, we use the methodology of Caswell (2009). We introduce a new state, FF*, into which mosquitoes enter immediately following a blood meal and remain for only one time step (i.e., there is no opportunity to remain in this state). The transitions (to *FF* or *HG*, or death) are otherwise exactly as for the *FF* state. The number of visits to FF* thus represents the number of blood meals taken over the course of a mosquitoes' life. To obtain a series of square matrices, we write a matrix ***M***_0_ = ***M***_*nd*_ ***M***_*d*_ ***M***_*dn*_. We assemble a block diagonal matrix U, with the individual periodic matrices ***M***_*i*_ on the diagonals (for 0 ≤ *i *≤* *12); a block diagonal matrix D, which has 13 × 13 matrices ***D*** along the diagonal, to switch between periods deterministically:

(7) $$D=\left[\begin{array}{ccccc} 0 & 0 & \ldots & 0 & 1\\ 1 & 0 & \ldots & 0 & 1\\ 0 & 1 & \ldots & 0 & 0\\ \vdots & \vdots & \ddots & \vdots & \vdots \\ 0 & 0 & \ldots & 0 & 0 \end{array}\right]$$

and a 143 × 143 vec‐permutation matrix ***K***_*s*,*q*_ (where subscripts *s* and *q* refer to the number of stages in the matrix and the number of periods, respectively). See Hunter and Caswell (2005) for details. These can be put together as a transition matrix where demographic transitions within periods and movement between periods is separated:

(8) $$\tilde{U}=DK_{s,q}UK_{s,q}^{T}.$$

From $\tilde{U}$ we can calculate the fundamental matrix $\tilde{N}$, which holds the expected number of visits to each stage per period, depending on an individual's initial period and stage:

(9) $$\tilde{N}={\left(I_{sq}-\tilde{U}\right)}^{-1}.$$

where ***I***_*sq*_ is the identity matrix. From $\tilde{N}$, we find the expected number of visits to FF*, for a newly emerged adult, assuming that emergence occurs at dusk.

## Results

**Figure 3:**
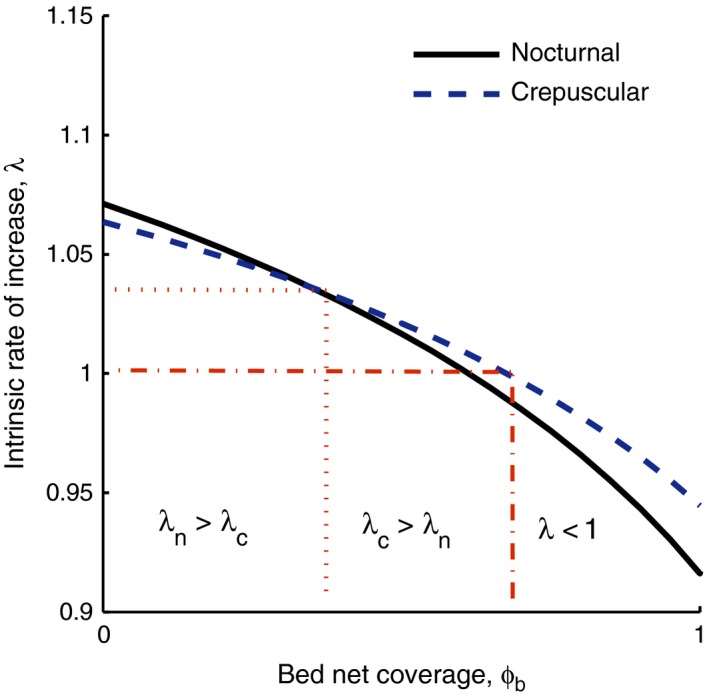
The intrinsic rate of increase, *λ*, a measure of fitness, for mosquitoes with a nocturnal (sensitive) biting pattern and mosquitoes with a crepuscular biting pattern (resistant), by coverage level of bed nets, $\phi_{b}$. The dashed and dotted lines divide the parameter space into levels of $\phi_{b}$ where the nocturnal pattern has a greater fitness than the crepuscular pattern (*λ*_*n*_ > *λ*_*c*_), an area of coverage where selection favors the crepuscular pattern (*λ*_*c*_ > *λ*_*n*_), and higher levels of coverage where *λ* of both types is below one and neither type will persist.

The fitness of mosquito populations, as measured by their intrinsic rate of increase, *λ*, is affected by the proportion of humans that are covered by (i.e., own and use) LLINs (Fig. 3). Three distinct outcomes are associated with increasing LLIN coverage. At low levels of coverage, the fitness of sensitive populations is greater than that of behaviorally resistant populations, and over time, the nocturnal type would persist. This fitness advantage is the result of the differences in foraging‐related mortality that we assumed to be associated with different times of the day (see Fig. 2). At intermediate LLIN coverage, the fitness of behaviorally resistant populations is greater than that of the nocturnal type. Here, over time, the resistant population would take over. While the rate at which resistance emerges will be a function of the difference in fitness between the two genotypes, it is important to note that the rate of increase for both sensitive and resistant populations decreases (albeit at different rates) with increasing LLIN coverage. Thus, as coverage reaches even higher levels, at some point, the rate of increase of both populations becomes smaller than one, and (barring immigration or a loss of bed net integrity) one expects the populations to go extinct.

**Figure 4:**
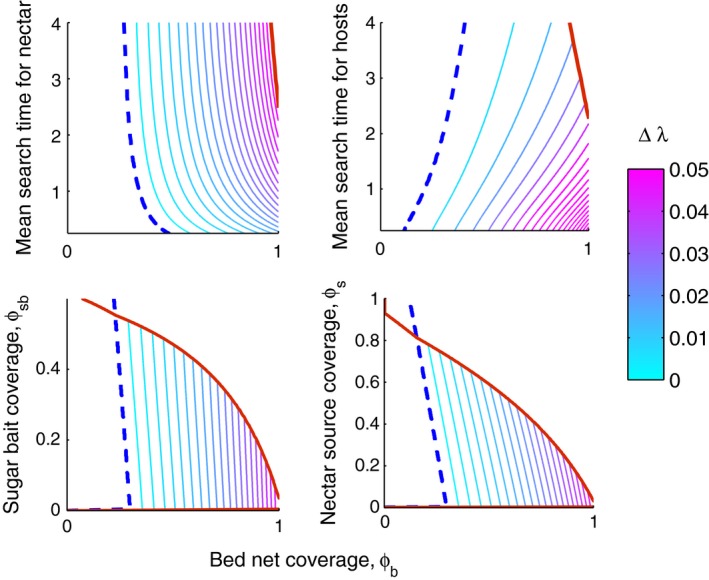
Fitness differences between nocturnal and crepuscular foraging patterns as a function of bed net coverage (horizontal axis) and four other environmental parameters (vertical axes). In each panel, the dashed line shows where the fitnesses of the two patterns are equal. To the left (right) of the dashed line, the fitness of the nocturnal forager is greater (less) than that of the crepuscular forager. To the right of the red line, *λ* of both types is <1. The area between the dashed blue and solid red lines gives the parameter space where behavioral resistance is favored. Within this space, the colored contour lines indicate the difference between *λ*_*n*_ and *λ*_*c*_. On their vertical axes, the panels show the effect of the expected searching time for nectar sources (in hours), 1/*γs* (upper left panel), the expected searching time for blood hosts (in hours), 1/*γb* (upper right panel); and sugar bait coverage (lower panels).

The exact coverage levels associated with these thresholds will depend on the parameter values used in the model. Here, we demonstrate how these thresholds may depend on environmental factors, and use them to explore the potential of integrated control options to mitigate the spread of resistance. We varied the parameter values related to two environmental aspects in our model: the encounter rates of nectar sources and those of blood hosts. Additionally, we varied LLIN coverage and explored the impact on fitness of adding ATSB alongside bed nets (Fig. 4). The latter was done using both approaches to sugar baits: addition of baits to the environment (‘sugar bait coverage') and replacement or spraying of existing nectar sources with toxic sugar (‘nectar source coverage').

Varying nectar source encounter rates, *γ*_*s*_, so that the mean search time for nectar varies from 10 min to 4 h reveals that sugar source abundance affects the fitness of sensitive and resistant populations in two relevant ways (Fig. 4, upper left panel). First, the threshold where behavioral resistance will emerge (the dashed line in Fig. 4) shifts to slightly greater levels of LLIN coverage as sugar becomes more abundant. Second, the threshold where the rate of increase of both types is below one likewise shifts to greater levels of LLIN use as sugar is more abundant (i.e., only when sugar is very rare is it possible for both nocturnal and crepuscular types to go extinct, even at very high levels of LLIN coverage).

Encounter rates with blood hosts, reflecting how densely or sparsely populated an area is, had a strong impact on the difference in fitness between nocturnal and crepuscular populations (Fig. 4, upper right panel). The threshold of extinction followed a similar, if more pronounced, pattern as it did for nectar sources: greater levels of LLIN coverage were required to eliminate mosquito populations as search time for hosts decreased. The LLIN coverage threshold where the fitness of resistant mosquitoes was greater than that of sensitive mosquitoes however, increased with higher host search times. Thus, blood host and nectar source encounter rates affect the emergence of resistance in different ways. A greater difference in fitness between sensitive and resistant populations, which may indicate a faster emergence of resistance, is found at high levels of blood host encounter rates (short search times for hosts), and at lower encounter rates of nectar sources (high search times for nectar).

From here, we shift focus to the potential of using a multipronged approach of bed nets and toxic sugar baits to vector and resistance management. We also investigate how the potential impact of an integrated approach depends on environmental conditions. Both approaches of ATSB gave qualitatively similar outcomes with regard to the emergence of resistance. Adding toxic sugar baits to the environment at varying levels of coverage led to two opposing effects (Fig. 4, lower panels). Elimination of vector populations becomes possible at increasingly lower rates of LLIN coverage, as toxic sugar bait coverage increases. If the threshold of elimination is not exceeded (i.e., the areas below the red lines), however, greater levels of ATSB coverage are associated with slightly greater differences in fitness between sensitive and resistant populations.

**Figure 5:**
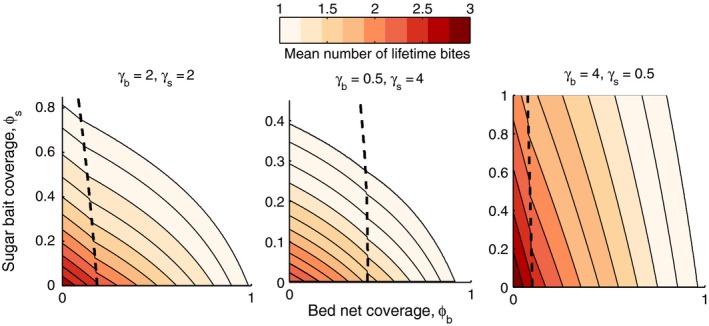
The effect of integrated control of bed nets and sugar baits at varying levels of coverage on the mean number of bites an individual mosquito will deliver, under conditions of moderate levels of nectar and blood hosts (left panel); high nectar encounter rates and low blood host encounter rates (center panel); and low nectar and high blood host encounter rates (right panel). The mean number of lifetime bites are plotted for nocturnal and crepuscular populations to the left and right of the dashed lines, respectively. The area where *λ* of both types is below one was not colored (there would be no transmission).

A resistance management strategy should not only slow down the emergence of resistance, but preferably do so without increasing the intensity of pathogen transmission. To investigate the impact of using ATSBs alongside LLINs on transmission potential, we varied both ATSB coverage (replacement of existing nectar sources with ATSB) and LLINs from 0 to 1. We then calculated the expected lifetime number of bites for individual mosquitoes (Fig. 5). We did this for three different combinations of encounter rates of blood hosts and nectar sources. We chose values to represent (i) moderate encounter rates for both blood and nectar sources; (ii) high encounter rates for blood and low encounter rates for nectar sources; and (iii) low encounter rates for blood and high encounter rates for nectar sources. The number of expected bites were evaluated for nocturnal populations where interventions resulted in *λ*_*n*_ being greater than *λ*_*c*_; for crepuscular populations where *λ*_*c*_ > *λ*_*n*_, and assumed to be zero where both populations went extinct. The expected number of bites of individual mosquitoes becomes smaller as LLIN coverage is increased. This occurs regardless of whether behavioral resistance emerges, although the rate of decline does decrease at the transition from nocturnal to crepuscular populations (Fig. 5). From a resistance management perspective, an important outcome is that LLIN coverage can in principle be decreased and supplemented with ATSB coverage to delay or prevent resistance from emerging, while not increasing the expected number of lifetime bites of mosquitoes. However, this does depend on the particular environmental conditions, for instance, in areas with low levels of nectar sources and abundant blood hosts trading off LLIN coverage for ATSB coverage would lead to an increase in the expected number of bites (Fig. 5, right panel).

**Figure 6:**
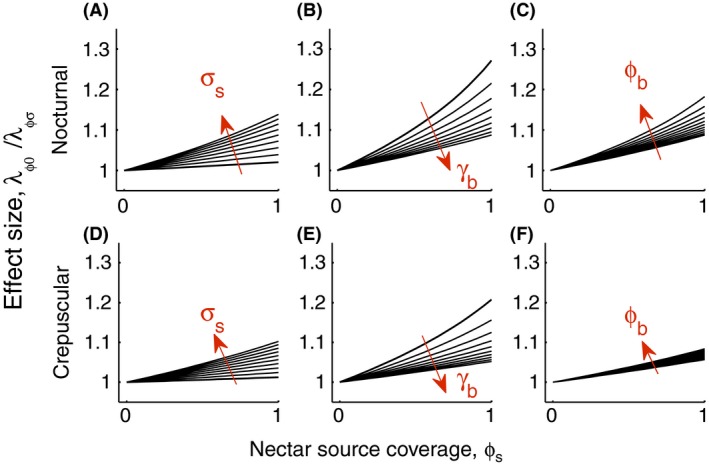
The effect size of toxic sugar bait coverage expressed as the rate of increase in the absence of sugar baits, $\lambda_{\phi_{0}}$, divided by the rate of increase at a given level of coverage, $\lambda_{\phi_{s}}$. Red arrows point in the direction of increasing values of the given parameter. The upper and lower rows depict the responses of nocturnal and crepuscular populations, respectively. The effect size of sugar baits increases with higher levels of nectar preference, *σ*_*s*_ (A, D); decreases with higher blood host encounter rates, *γ*_*b*_ (B, E); and increases with bed net coverage rates, $\phi_{b}$ (C, F).

In reality, vector control interventions can also affect population densities. While our current modeling approach is not amenable to incorporation of seasonality or a carrying capacity, we can assume that decreasing the rate of increase of a population in a realistic seasonal environment would slow down the rate at which the carrying capacity is approached, and thereby potentially the average density of vectors over a course of a season. To assess the potential efficacy of the secondary control method (ATSB, where again a proportion of sugar sources are assumed to be toxic) in the context of an integrated approach on population suppression, we calculated an effect size that quantifies the effect of ATSBs on mosquito population growth. Specifically, we calculated the rate of increase of the population in the absence of an ATSB intervention, divided by the rate of increase at a given level of that intervention. We calculated this effect size under varying conditions by varying several parameter values, including the tendency of the vector species to accept a sugar meal (*σ*_*s*_); the encounter rate of blood hosts (*γ*_*b*_); and the population coverage of LLINs $(\phi_{b}).$ This was carried out for both nocturnal and crepuscular populations (Fig. 6, upper and lower panels, respectively). The results suggest an obvious point: the efficacy of sugar baits, and the extent to which they will be useful in specific situations, is dependent on the frequency with which mosquitoes make use of nectar as a resource. As the tendency of mosquitoes to accept a sugar meal when encountered increases, so does the efficacy of sugar baits. As encounter rates with blood hosts increase, there is a diminishing efficacy of sugar baits, because mosquitoes are now more likely to forego sugar feeding. With increasing rates of LLIN coverage, access to blood hosts is more restricted, and the efficacy of sugar baits increases. These patterns are largely similar for nocturnal and crepuscular populations, although the synergistic effect between LLINs and sugar baits is less strong in the case of behaviorally resistant mosquitoes.

## Discussion

Environmental factors may play a key part in molding the selection pressures placed on mosquito populations by insecticide‐based control interventions. Typically, environmental stressors appear to either exacerbate fitness costs associated with physiological resistance mechanisms, or weaken the fitness advantage associated with resistance in the presence of the insecticide (Raymond et al. 2005; Farenhorst et al. 2009; Koella et al. 2012). In the case of behavioral resistance, trade‐offs affecting the expression of costs and benefits of resistance may have less to do with physiological mechanisms, but rather depend on host and vector behavioral patterns, opportunity costs, and risks associated with foraging. It appears that environmental stressors related to foraging (encounter rates, which may translate as waiting times until blood or nectar meals are obtained) have opposing effects on the evolution of behavioral resistance. Namely increased access to blood hosts lowers the threshold of LLIN coverage at which resistance emerges, while increased encounter rates with nectar sources raises this threshold.

These results have several practical implications for malaria control. One promising area is whether resource encounter rates can help predict whether or not behavioral resistance is likely to emerge. For instance, the model predicted that behavioral resistance will emerge most rapidly in densely populated areas with high coverage levels of LLINs (as suggested by the greater difference in fitness between nocturnal and crepuscular populations). In more sparsely populated areas, the range of LLIN coverage levels that leads to resistance is narrower, and resistance would emerge more slowly. Higher encounter rates with nectar sources slightly increased the LLIN coverage threshold at which both nocturnal and crepuscular populations would go extinct. Thus, even at near universal levels of LLIN coverage, behaviorally resistant populations may be likely to persist and potentially continue to transmit disease, unless they are in areas with very high search times for nectar sources.

There was a modest effect of encounter rates with sugar on the threshold at which resistance emerged. A plausible explanation is that abundant nectar sources allowed nocturnally activating mosquitoes more behavioral plasticity in the presence of bed nets by letting them meet their energetic needs and survive until dawn or the next evening. Although this effect is unlikely to predict whether resistance will emerge, the rate at which resistance emerges would be lower in areas with highly abundant sugar sources. Such an effect of plasticity on evolution is well known (Stearns 1989; Robinson and Dukas 1999), although it is worth considering whether this model truly captures phenotypic plasticity (environmentally induced changes in phenotypes). If we denote the phenotype as the foraging strategy (nocturnal or crepuscular activation patterns), then our model does not incorporate this per se. If we denote the distribution of biting times as the phenotype, then our model does capture phenotypic plasticity. Regardless, we note that this (apparent) behavioral plasticity can redistribute biting times somewhat, sufficiently to have an impact on the emergence of resistance, but not so much so that it would by itself explain the large shifts in biting times that have been observed in some situations. This corresponds to recent findings from another modeling study (Killeen and Chitnis 2014). Shifts in periodicity could also be due to associative learning on the part of the vectors, although evidence for this is lacking. Further, we assumed that there are only two distinct genotypes associated with diel biting patterns: nocturnal and crepuscular ones. It is possible that, instead, there are many intermediate steps and this behavior is under the control of many genes. In this model, there was also no interaction between genotypes, but if greater numbers of early biting mosquitoes were to have an influence on host behavior [e.g., make it more likely that they will use a bed net that night, or become more defensive (Kelly and Thompson 2000)], then the fitness costs and benefits of behavioral resistance could be density dependent. Further studies on the genetic and/or behavioral basis of this type of resistance would be valuable, and allow for models to be developed that explicitly include genetic structure and thereby allow for predictions regarding the rate at which resistance could spread.

Our results suggest behavioral resistance would not lead to a failure of LLINs as a control method. This result needs to be interpreted with care, because the metric for disease transmission we were able to use for this model, the expected number of lifetime bites per female, only captures part of the important transmission parameters. Given this limitation, our results suggest that even if a population tends to forage in the crepuscular period, increasing LLIN coverage still decreases the number of lifetime bites and fitness of such populations, even if crepuscular populations persist at higher LLIN coverages than do nocturnal ones. A practical implication is that simply increasing LLIN coverage could potentially mitigate this type of resistance. However, the coverage and compliance levels required (in some areas, near universal) may stretch the capacity of vector control programs, and also assumes LLINs will not degrade over time. An integrated vector management approach where ATSB are used along with LLINs may therefore be appealing and allows for two possible strategies. The first approach would be to not attempt to delay resistance, but aim to control resistant populations once they have arisen, that is, maintain LLIN coverage, but add a supplementary measure to further suppress transmission or even target vector elimination at a local scale (Killeen 2014). This approach is predicated on the ability to add interventions while maintaining LLIN coverage. In resource‐constrained settings, where increasing coverage or adding an intervention may not always be feasible, a second option is to use an integrated approach of lower LLIN levels with ATSB coverage, thereby preventing or delaying the spread of behavioral resistance. This could be particularly appealing if it were to work as a rotation‐based strategy. We found that a synergistic effect between LLINs and ATSB was greater when mosquito populations were of the nocturnal type. It is possible then that an integrated approach that does delay resistance will be more (cost‐)effective in the long run.

The efficacy of such an integrated vector management approach using LLINs and ATSB also depends on local environmental conditions. For instance, the extent to which delaying resistance was a feasible strategy depended on the abundance of both sugar sources and blood hosts (Fig. 5). When sugar sources were rare (and note that here we were replacing or spraying existing nectar sources, rather than adding new sources (baits) to the environment) and blood hosts abundant, delaying the emergence of resistance while reducing the expected number of bites per mosquito to the same extent was not feasible. The impact of environmental factors on the effect size of ATSB (Fig. 6) illustrates that both the acceptability of local nectar sources and the abundance of blood hosts will determine the efficacy of an integrated approach using toxic sugar baits.

We were able to highlight the facultative (i.e., dependent on blood host availability) nature of sugar feeding using a state‐based model and treating sugar‐feeding behavior as a component of the foraging process. This provides an example where capturing vector behavior in models in a more realistic manner than is typical of Ross‐Macdonald style models (Smith et al. 2012) is relevant. A disadvantage is that many parameters are often not measured at the fine scale required here (e.g., estimates of survival are more likely to be available at a daily level than at an hourly level for various physiological and behavioral states). Before models such as these could be used in a truly predictive fashion, further entomological work will therefore be required to allow for a more careful parameterization. Extensions such as the inclusion of density‐dependent processes related to larval development and mortality, seasonality, and genetic structure would likewise be required, and other simplifications we made regarding the biology of malaria vectors may also have to be addressed in a more ambitious model. For instance, while we assumed mosquitoes would only feed on sugar while unfed, females in reality may take sugar throughout the gonotrophic cycle, and depending on oviposition site restrictions, they may particularly increase their use of this resource while in the gravid state (Gary and Foster 2006). This should not affect our results as we did not vary larval habitat abundance, but for a model investigating a combination of ATSB and larval source management, such detail could be relevant. Additionally, sugar feeding may be expressed more or less strongly depending on age (Foster and Takken 2004), have a different diel periodicity than blood host seeking, or depend on the extent of spatial separation between domiciles and nectariferous plants and the outside predation risk (Ma and Roitberg 2008; Roitberg and Mangel 2010). Individual‐ or agent‐based models that track energetic state to capture state‐based foraging decisions in more detail do exist (Ma and Roitberg 2008; Roitberg and Mangel 2010; Zhu et al. 2015). However, our simplified behavioral model captures two critical aspects of sugar‐feeding behavior: (i) sugar feeding tends to occur facultatively, that is, it becomes more common as blood hosts are rarer and (ii) it affects vectorial capacity by both extending the duration of the gonotrophic cycle and lowering the biting rate on humans, and increasing mosquito survivorship (Stone and Foster 2013). We also did not include infection of mosquitoes here, but female mosquitoes infected with *Plasmodium* may be more attracted to nectar sources (Nyasembe et al. 2014), presumably further increasing the efficacy of ATSB as a malaria control method.

We also did not explicitly account for biting on non‐human blood hosts. Instead, we assumed that non‐humans could be considered as systematic noncompliers of bed nets, given our interest in fitness rather than pathogen transmission. By that logic, a lower value of bed net coverage, *θ*_*b*_, could also be interpreted as a higher availability of non‐human hosts. This would suggest that in situations with higher abundances of non‐humans (and a vector that is opportunistic in its biting behavior, such as *An. arabiensis*), the evolution of behavioral resistance would be delayed. However, this would need to be investigated more carefully, as other parameters (the encounter rate of hosts, *γ*_*b*_, and possibly the preference for hosts, *σ*_*b*_) would also change.

We focused on the usefulness of using LLINs in combination with ATSB as an example of an integrated approach. Combinations of different control methods may be more efficacious in some environments. Further, we focused solely on the evolution of resistance in one relatively narrow sense: a shift in peak biting times, and did not consider other potentially adaptive responses. These could include changing preferences for host types (Lyimo et al. 2013), minimizing the time spent in contact with insecticide‐treated surfaces (Killeen 2014), avoiding sugar baits (Wada‐Katsumata et al. 2013), or perhaps most importantly, developing physiological resistance. When devising an integrated management approach, one would have to be cognizant of other such outcomes. This suggests that resistance or integrated vector management programs will have to be tailored to specific areas, and will be maximally effective if knowledge of local ecological and environmental factors can be accounted for in their design. A potential drawback currently is that our understanding of the selection pressures that act on these various adaptive responses in different local environments remains scant.

In conclusion, we have shown that environmental factors, most strongly encounter rates with blood hosts, can affect the rate at which behavioral resistance emerges. An intriguing consideration is whether this may help explain why the evidence for shifts in mosquito biting times remains equivocal. If these findings were to be confirmed under field conditions, we may not only be able to better predict the type of vector population‐level response to control interventions, but also be able to use this information to design the most (cost‐)effective control methods.

## Appendix

The transition matrices that project from one hour to the next during the nighttime, ***M***_*i*_ are 10 × 10 matrices given by:

(10) $$\begin{array}{cc} & M_{\left(i\right)}=\\ & \left[\begin{array}{cccccccccc} t_{1,1} & 0 & 0 & 0 & 0 & 0 & 0 & 0 & 0 & t_{1,10}\\ t_{2,1} & t_{2,2} & 0 & 0 & 0 & 0 & 0 & 0 & 0 & t_{2,10}\\ 0 & t_{3,2} & t_{3,3} & 0 & 0 & 0 & 0 & 0 & 0 & 0\\ 0 & 0 & t_{4,3} & t_{4,4} & 0 & 0 & 0 & 0 & 0 & 0\\ 0 & 0 & 0 & t_{5,4} & t_{5,5} & 0 & 0 & 0 & 0 & 0\\ 0 & 0 & t_{6,3} & 0 & t_{6,5} & t_{6,6} & 0 & 0 & 0 & 0\\ 0 & 0 & 0 & 0 & 0 & t_{7,6} & t_{7,7} & 0 & 0 & 0\\ 0 & 0 & 0 & 0 & 0 & 0 & t_{8,7} & t_{8,8} & 0 & 0\\ 0 & 0 & 0 & 0 & 0 & 0 & 0 & t_{9,8} & t_{9,9} & 0\\ 0 & 0 & 0 & 0 & 0 & 0 & 0 & 0 & t_{10,9} & t_{10,10} \end{array}\right] \end{array}$$

To transition to the daytime period, during which we assume mosquitoes only undergo physiological but no behavioral processes, requires a matrix to project from the final hour in the nighttime (around dawn) to day:

(11) $$M_{\left(dn\right)}=\left[\begin{array}{cccccccccc} 1 & 0 & 0 & 0 & 0 & 0 & 0 & 0 & 0 & 0\\ 0 & 1 & 0 & 0 & 0 & 0 & 0 & 0 & 0 & 0\\ 0 & 0 & 1 & 0 & 0 & 0 & 0 & 0 & 0 & 0\\ 0 & 0 & 0 & 1 & 0 & 0 & 0 & 0 & 0 & 0\\ 0 & 0 & 0 & 0 & 1 & 0 & 0 & 0 & 0 & 0\\ 0 & 0 & 0 & 0 & 0 & 1 & 0 & 0 & 0 & 0\\ 0 & 0 & 0 & 0 & 0 & 0 & 1 & 0 & 0 & 0\\ 0 & 0 & 0 & 0 & 0 & 0 & 0 & 1 & 0 & 0\\ 0 & 0 & 0 & 0 & 0 & 0 & 0 & 0 & 1 & 1 \end{array}\right]$$

From here, as little happens during the day, this period will be covered in a single, 12‐h‐long time step.

(12) $$\begin{array}{cc} & M_{\left(d\right)}=\\ & \left[\begin{array}{ccccccccc} \alpha_{1,1} & 0 & 0 & 0 & 0 & 0 & 0 & 0 & 0\\ \alpha_{2,1} & \alpha_{2,2} & 0 & \alpha_{2,4} & 0 & 0 & 0 & 0 & 0\\ 0 & 0 & \alpha_{3,3} & 0 & \alpha_{3,5} & 0 & 0 & 0 & 0\\ 0 & 0 & 0 & \alpha_{4,4} & 0 & 0 & 0 & 0 & 0\\ 0 & 0 & 0 & 0 & \alpha_{5,5} & 0 & 0 & 0 & 0\\ 0 & 0 & 0 & 0 & 0 & \alpha_{6,6} & 0 & 0 & 0\\ 0 & 0 & 0 & 0 & 0 & \alpha_{7,6} & \alpha_{7,7} & 0 & 0\\ 0 & 0 & 0 & 0 & 0 & 0 & \alpha_{8,7} & \alpha_{8,8} & 0\\ 0 & 0 & 0 & 0 & 0 & 0 & 0 & \alpha_{9,8} & \alpha_{9,9} \end{array}\right] \end{array}$$

The only transitions, indicated here by *α*_*ij*_ instead of *t*_*ij*_ to highlight the different time step involved, that therefore occur during the day are the development and emergence of adult mosquitoes and gonotrophic development. The transitions *α*_*ij*_ and *t*_*ij*_ are defined below.The matrix *M*_(*nd*)_ projects the daytime physiological stages back into the stages associated with nighttime:

(13) $$M_{\left(nd\right)}=\left[\begin{array}{ccccccccc} 1 & 0 & 0 & 0 & 0 & 0 & 0 & 0 & 0\\ 0 & 1 & 0 & 0 & 0 & 0 & 0 & 0 & 0\\ 0 & 0 & 1 & 0 & 0 & 0 & 0 & 0 & 0\\ 0 & 0 & 0 & 1 & 0 & 0 & 0 & 0 & 0\\ 0 & 0 & 0 & 0 & 1 & 0 & 0 & 0 & 0\\ 0 & 0 & 0 & 0 & 0 & 1 & 0 & 0 & 0\\ 0 & 0 & 0 & 0 & 0 & 0 & 1 & 0 & 0\\ 0 & 0 & 0 & 0 & 0 & 0 & 0 & 1 & 0\\ 0 & 0 & 0 & 0 & 0 & 0 & 0 & 0 & 1\\ 0 & 0 & 0 & 0 & 0 & 0 & 0 & 0 & 0 \end{array}\right]$$

The matrix transition elements are as follows, where for each stage the sum of the probabilities of remaining in or progressing to a different stage sum to less than one, and the remainder corresponds to mortality acting on that stage. For rates where time is not specifically indicated, a one‐hour time step is used:

(14) $$t_{1,1}=e^{-\frac{1}{\varepsilon }}e^{-\mu_{l}},$$

(15) $$t_{2,1}=\left(1-e^{-\frac{1}{\varepsilon }}\right)e^{-\mu_{l}},$$

(16) $$t_{2,2}=e^{-\mu_{uf}}\left(1-\rho_{i}\right),$$

(17) $$t_{3,2}=\rho_{i}e^{-\mu_{uf}},$$

(18) $$t_{3,3}=e^{-\mu_{f,i}}e^{-(\gamma_{b}\sigma_{b}+\gamma_{s}\sigma_{s})},$$

(19) $$t_{4,3}=e^{-\mu_{f,i}}\left(1-e^{-(\gamma_{b}\sigma_{b}+\gamma_{s}\sigma_{s})}\right)a_{s},$$

(20) $$t_{6,3}=e^{-\mu_{f,i}}\left(1-e^{-(\gamma_{b}\sigma_{b}+\gamma_{s}\sigma_{s})}\right)a_{b},$$

(21) $$t_{4,4}=e^{-\mu_{sf}}\left(1-\rho_{i}\right),$$

(22) $$t_{5,4}=e^{-\mu_{sf}}\rho_{i},$$

(23) $$t_{5,5}=e^{-\mu_{f_{s},i}}e^{-(\gamma_{b}\sigma_{b})},$$

(24) $$t_{6,5}=e^{-\mu_{f_{s},i}}\left(1-e^{-(\gamma_{b}\sigma_{b})}\right),$$

(25) $$t_{6,6}=t_{7,7}=t_{8,8}=e^{-\mu_{r}}e^{-\frac{1}{\delta_{b}}},$$

(26) $$t_{7,6}=t_{8,7}=t_{9,8}=e^{-\mu_{r}}\left(1-e^{-\frac{1}{\delta_{b}}}\right),$$

(27) $$t_{9,9}=e^{-\mu_{r}}\left(1-\rho_{o}\right),$$

(28) $$t_{10,9}=e^{-\mu_{r}}\rho_{o},$$

(29) $$t_{10,10}=e^{-\mu_{f}}e^{-\gamma_{o}},$$

(30) $$t_{2,10}=\left(1-e^{\gamma_{o}}\right)e^{-\mu_{f}},$$

(31) $$t_{1,10}=\left(1-e^{\gamma_{o}}\right)e^{-\mu_{f}}\frac{1}{2}F,$$

and for the day to night matrix:

(32) $$\alpha_{1,1}=e^{-(\frac{1}{\epsilon }\times 12\;h)}e^{-(\mu_{l}\times 12\;h)},$$

(33) $$\alpha_{2,1}=\left(1-e^{-\frac{1}{\epsilon }\times 12\;h}\right)e^{-(\mu_{l}\times 12\;h)},$$

(34) $$\alpha_{2,2}=e^{-(\mu_{uf}\times 12\;h)},$$

(35) $$\alpha_{3,3}=e^{-(\mu_{uf}\times 12\;h)},$$

(36) $$\alpha_{4,4}=e^{-(\mu_{sf}\times 12\;h)}e^{-\frac{1}{\delta_{s}}\times 12\;h},$$

(37) $$\alpha_{2,4}=e^{-(\mu_{sf}\times 12\;h)}\left(1-e^{-\frac{1}{\delta_{s}}\times 12\;h}\right),$$

(38) $$\alpha_{5,5}=e^{-(\mu_{sf}\times 12\;h)}e^{-\frac{1}{\delta_{s}}\times 12\;h},$$

(39) $$\alpha_{3,5}=e^{-(\mu_{sf}\times 12\;h)}\left(1-e^{-\frac{1}{\delta_{s}}\times 12\;h}\right),$$

(40) $$\alpha_{6,6}=\alpha_{7,7}=\alpha_{8,8}=e^{-\frac{1}{\delta_{b}}\times 12\;h}e^{-(\mu_{r}\times 12\;h)},$$

(41) $$\alpha_{7,6}=\alpha_{8,7}=\alpha_{9,8}=\left(1-e^{-\frac{1}{\delta_{b}}\times 12\;h}\right)e^{-(\mu_{r}\times 12\;h)},$$

(42) $$\alpha_{9,9}=e^{-(\mu_{r}\times 12\;h)}.$$

Certain transitions are expanded upon to capture the effects of vector control methods. The transition probability from host seeking to freshly fed, in the presence of LLINs becomes,

(43) $$t_{6,3}=e^{-\mu_{f,i}}\left(1-e^{-(\gamma_{b}\sigma_{b}+\gamma_{s}\sigma_{s})}\right)a_{b}\left(1-\phi_{b,i}\right).$$

While for sugar‐fed host‐seeking mosquitoes this becomes,

(44) $$t_{6,5}=e^{-\mu_{f_{s},i}}\left(1-e^{-\gamma_{b}\sigma_{b}}\right)\left(1-\phi_{b,i}\right).$$

The transitions that determine whether a mosquito remains in the host‐seeking state become:

(45) $$t_{3,3}=e^{-\mu_{f,i}}e^{-(\gamma_{b}\sigma_{b}+\gamma_{s}\sigma_{s})}+e^{-\mu_{f,i}}\left(1-e^{-(\gamma_{b}\sigma_{b}+\gamma_{s}\sigma_{s})}\right)a_{b}\phi_{b,i}r,$$

(46) $$t_{5,5}=e^{-\mu_{f_{s},i}}e^{-(\gamma_{b}\sigma_{b})}+e^{-\mu_{f,i}}\left(1-e^{-(\gamma_{b}\sigma_{b})}\right)\phi_{b,i}r_{s}.$$

To incorporate ATSB, when we assume bait stations are added to the environment, we only change one transition,

(47) $$t_{4,3}=e^{-\mu_{f,i}}\left(1-e^{-(\gamma_{b}\sigma_{b}+\gamma_{s_{b}}\sigma_{s_{b}}+\gamma_{s_{n}}\sigma_{s_{n}})}\right)a_{s_{n}},$$

where $a_{s_{n}}=\frac{\gamma_{s_{n}}\sigma_{s_{n}}}{\gamma_{b}\sigma_{b}+\gamma_{s_{n}}\sigma_{s_{n}}+\gamma_{s_{b}}\sigma_{s_{b}}}$. While for the alternative method, where existing nectar sources are sprayed with toxic sugar:

(48) $$t_{4,3}=e^{-\mu_{f,i}}\left(1-e^{-(\gamma_{b}\sigma_{b}+\gamma_{s}\sigma_{s})}\right)a_{s}\left(1-\phi_{s}\right).$$
